# Macrophage PKM2 depletion ameliorates hepatic inflammation and acute liver injury in mice

**DOI:** 10.3389/fphar.2025.1546045

**Published:** 2025-04-25

**Authors:** Ziwei Kang, Ruoyan Xie, Yiming Cui, Zhiwei Chen, Jincheng Li, Jinyu Lv, Weijia Ye, Peixin Zhao, Keke Zhang, Jian Hong, Hengdong Qu

**Affiliations:** ^1^ State Key Laboratory of Bioactive Molecules and Druggability Assessment, Jinan University, Guangzhou, China; ^2^ Department of Pathophysiology, School of Medicine, Jinan University, Guangzhou, China; ^3^ Department of Hepatobiliary Surgery, The First Affiliated Hospital, Jinan University, Guangzhou, China; ^4^ Center of Hepato-Pancreato-Biliary Surgery, The First Affiliated Hospital, Sun Yat-sen University, Guangzhou, China

**Keywords:** pyruvate kinase M2 (PKM2), macrophages, glycolysis, hepatocyte, acute liver injury (ALI)

## Abstract

**Introduction:**

Pyruvate kinase M2 (PKM2), the rate-limiting enzyme of glycolysis, plays a critical role in macrophage activation and a broad spectrum of chronic liver diseases. However, whether PKM2 contributes to the pathogenesis of acute liver injury (ALI) remains largely unexplored.

**Methods:**

PKM2 expression was assessed in human and mouse ALI livers. Macrophage-specific PKM2 knockout mice were challenged by two independent ALI models, induced by acetaminophen (APAP) and lipopolysaccharide/D-galactosamine (LPS/D-GalN), to explore the role and regulatory mechanism of macrophage PKM2 in ALI progression.

**Results:**

By bioinformatic screening and analysis of ALI liver, we found that PKM2 was significantly upregulated in the liver tissues of ALI patients and mice. Immunofluorescence staining further demonstrated that PKM2 was markedly upregulated in macrophages during ALI progression. Notably, macrophage PKM2 depletion effectively alleviated APAP- and LPS/D-GalN-induced ALI, as demonstrated by ameliorated immune cells infiltration, pro-inflammatory mediators, and hepatocellular cell death. PKM2-deficient macrophages showed M2 anti-inflammatory polarization *in vivo* and *in vitro*. Furthermore, PKM2 deletion limited HIF-1α signaling and aerobic glycolysis of macrophages, which thereby attenuated macrophage pro-inflammatory activation and hepatocyte injury. Pharmacological PKM2 antagonist efficiently ameliorated liver injury and prolonged the survival of mice in APAP-induced ALI model.

**Discussion:**

Our study highlights the pivotal role of macrophage PKM2 in advancing ALI, and therapeutic targeting of PKM2 may serve as a novel strategy to combat ALI.

## 1 Introduction

Liver, the largest solid organ of the body, shows remarkable capacity against drug toxicity and bacterial/viral infection. Acute liver injury (ALI), characterized by innate immunity dysregulation and overwhelming hepatocellular cell death, is a fatal liver condition with high morbidity and mortality (Stravitz and Lee, 2019). Acetaminophen (APAP) overdose is a leading cause of drug-induced ALI and acute liver failure (ALF) in the western world, accounting for nearly 50% of all the ALF cases in the United States (Bhushan and Apte, 2019; Katarey and Verma, 2016). Meanwhile, sepsis is another well-known cause of ALI, which has been recognized as a powerful independent predictor of mortality in the intensive care unit (Sun et al., 2020). Thus far, limited therapeutic options were available for ALI.

Myeloid cell infiltration is central to the pathogenesis of ALI, leading to enhanced hepatic inflammation and necrosis of centrilobular areas. Macrophage, an important component of the myeloid profiling, play a key role in the initiation, propagation, and finally the resolution of liver injury (Hassan et al., 2023; Roth et al., 2020). On one hand, in response to pro-inflammatory stimuli, macrophages release substantial oxygen free radicals and pro-inflammatory mediators, including interleukin-6 (IL-6) and tumor necrosis factor α (TNF-α), which triggers hepatocyte injury and apoptosis (Brenner et al., 2013). On the other hand, restorative macrophages are thought to orchestrate the clearance of DAMPs or PAMPs by phagocytosis, and thereby ameliorating hepatocyte apoptosis and liver injury (Li et al., 2020; Starkey Lewis et al., 2020). Recent evidences have highlighted the therapeutic potential of repolarizing macrophage from M1 to M2 phenotype as ALI treatment (Zhou et al., 2022; Yang et al., 2023). Further understanding of the macrophage phenotype switch may open up new avenues of macrophage-based therapeutic strategies for ALI.

Pyruvate kinase M2 (PKM2), the rate-limiting enzyme of glycolysis, has been recognized as a pivotal regulator of macrophage pro-inflammatory activation (Liu et al., 2021). Nuclear translocation of PKM2 induces transactivation of HIF-1α signaling and macrophage M1 polarization, whereas limiting PKM2 nuclear translocation by PKM2 antagonist reprograms macrophage from pro-inflammatory phenotype to restorative phenotype (Palsson-McDermott et al., 2015). Thus far, therapeutic targeting of PKM2 has been shown to ameliorate a broad spectrum of chronic liver diseases, ranging from alcoholic liver disease to nonalcoholic steatohepatitis (NASH) (Qu et al., 2023). Recent studies (Wang et al., 2022a; Sang et al., 2024) indicates that PKM2 is involved in the progression of ALI, however, the role and regulatory mechanism of PKM2 in ALI remains incompletely understood.

In the current study, we found that PKM2 was significantly upregulated in macrophages of ALI liver, and deletion of PKM2 in macrophages markedly ameliorated APAP- and LPS/GalN-induced ALI in mice. In addition, we found that therapeutic targeting of PKM2 efficiently alleviated liver injury and prolonged the survival of APAP mice. Our study demonstrates a central role of macrophage PKM2 in the progression of ALI, highlighting the translational potential of PKM2 as a therapeutic target for ALI treatment.

## 2 Methods

### 2.1 Bioinformatic analysis

Publicly available dataset (GSE255777) were used for analysis of *PKM* mRNA levels in human ALF, and the dataset regarding *PKM2* ^*ΔMAC*^ and *PKM2* ^*FL/FL*^ BMDMs were generated as previously described (Qu et al., 2024). Significant pathways of the differentially expressed genes (DEGs) were analyzed according to the Kyoto Encyclopedia of Genes and Genomes (KEGG) database.

### 2.2 Mice and treatment

Studies were performed on mice kept in a specific pathogen–free environment with a 12-h light-dark cycle starting at 7 a.m. at a constant temperature of 22°C–24°C. Macrophage-specific PKM2 knockout mice [*PKM2* ^*FL/FL*^ *LysM-Cre* (*PKM2* ^*ΔMAC*^)] and their control littermates (*PKM2* ^*FL/FL*^) were generated and validated as previous described (Qu et al., 2024). 6-8-week-old male C57BL/6J mice were obtained from Vital River Laboratory Animal Technology Co., Ltd. For all APAP experiments, mice were fasted overnight, and APAP (Sangon Biotech, Shanghai, China) was dissolved in warm 0.9% saline and was administered intraperitoneally (i.p.).

For analysis of the dynamic expression of macrophage PKM2 during APAP-induced ALI progression, mice were treated with 300 mg/kg APAP, and euthanized at 0, 6, 12, 24 h following APAP treatment. To interrogate the role of macrophage PKM2 in APAP-induced ALI progression, 6-8-week-old male *PKM2* ^*ΔMAC*^ and *PKM2* ^*FL/FL*^ mice were subjected to APAP challenge (300 mg/kg) and were euthanized and analyzed 24 h after induction. To determine the effect of macrophage PKM2 knockout on sepsis-associated ALI, 6-8-week-old male *PKM2* ^*ΔMAC*^ and *PKM2* ^*FL/FL*^ mice were subjected to 50 μg/kg LPS (Aladdin, Shanghai, China) plus 700 mg/kg D-GalN (Aladdin, Shanghai, China) and were euthanized and analyzed 5 h after induction. To investigate the therapeutic effect of PKM2 antagonist on APAP-induced ALI and ALF, 6-8-week-old male C57BL/6J mice were pretreated with single i.p. injection of ML265 (30 mg/kg; MedChemExpress, USA), and then were subjected to 300 mg/kg or 750 mg/kg APAP induction, for analysis of liver injury and survival respectively.

All animal procedures were performed in accordance with the Guidelines for Care and Use of Laboratory Animals of Jinan University and approved by the Animal Ethics Committee of Jinan University (The animal ethical license No. 20241112-04).

### 2.3 Histology, immunohistochemistry, and immunofluorescence

Briefly, formalin-fixed, paraffin-embedded liver tissue samples (4 μm) were stained with H&E, immunohistochemistry and immunofluorescence according to standard protocols as previously described (Qu et al., 2024). All bright field images were captured by Olympus BX51 Fluorescence Microscope. For immunofluorescence staining, sections of formalin-fixed, paraffin-embedded tissue samples or cells fixed with cold methanol were co-stained with indicated primary antibodies and further detected by appropriate secondary antibodies (ThermoFisher Scientific, MA, USA) labeled with either Alexa 488 or Alexa 555 according to the manufacturer’s instructions. DAPI (ThermoFisher Scientific) was used to label the nuclei. Immunofluorescence images were captured by Leica SP8 3X STED Laser Confocal Microscope. Morphologically, necrosis is identified as cellular swelling and chromatin condensation, and necrotic region was analyzed as previously described (Bi et al., 2022). Quantification of immunohistochemistry and immunofluorescence staining were performed by ImageJ as previously described (Qu et al., 2024). The following primary antibodies, including PKM1 (7067, CST), PKM2 (4053, CST), HNF-4α (3113, CST), F4/80 (70076, CST), CD11b (BM3925, Boster), C-Caspase3 (9664, CST), iNOS (F01770, Selleck), CD206 (24595, CST) were used for IHC and IF staining.

### 2.4 TUNEL

Mouse liver paraffin sections were stained with transferase-mediated deoxyuridine triphosphate-biotin nick end labeling (TUNEL) kit (Beyotime, Shanghai, China) ac-cording to the instructions.

### 2.5 Transfection

Lentiviral particles containing shPKM2 were obtained from GeneChem as previously described (Qu et al., 2024). Specific siRNAs targeting HIF-1α were designed and constructed by Genscript, and cells were transiently transfected with Lipofectamine 3000. Cells were used 48–72 h after transfection for downstream analyses. The sequences (5′-3′) are provided as below. siRNA-1: CAC​AUU​CAC​GUA​UAU​GAU​AUU; siRNA-2: CCG​GUU​GAA​UCU​UCA​GAU​AUU; siRNA-3: GUG​GAU​AGU​GAU​AUG​GUC​AUU.

### 2.6 Primary cell isolation and cell culture

Mouse bone marrow was collected from the femoral and tibia of mice and cultured in 10 ng/mL M-CSF (315-02, Peprotech, NJ, USA) containing DMEM supplemented with 10% FBS for 7 days for BMDMs differentiation. BMDMs were primed with LPS (10 ng/mL, Sigma-Aldrich) and IFN-γ (25 ng/mL, Peprotech) for 16 h to induce macrophage pro-inflammatory phenotype. Addition of 2-DG (S4701, Selleckchem, Texas, USA) were added to pre-treat the cells before LPS induction as previously described (Qu et al., 2024; Xie et al., 2016).

Primary hepatocytes were isolated as previously described (Bi et al., 2022; Wang et al., 2022b). Hepatocytes were cultured with DMEM medium, containing FBS, dexamethasone, insulin and penicillin-streptomycin solution. Thereafter, hepatocyte was co-cultured with BMDMs in the presence of LPS and IFN-γ, and the levels of pro-inflammatory factors of hepatocyte were determined by qPCR and ELISA. Meanwhile, the protein expression of p-STAT1 and HIF-1α in BMDMs was assessed by Western blotting.

The immortalized human myeloid leukemia monocytes (THP-1) cell line was purchased from Wuhan Procell Life Science & Technology Co., Ltd. (China) and cultured in RMPI medium supplemented with 10% FBS and 1% penicillin/streptomycin. The immortalized human hepatocytes (HepaRG) cell line was purchased from Shanghai Qiansi Biotechnology Co., Ltd. (China) and cultured in DMEM medium supplemented with 10% FBS and 1% penicillin/streptomycin. For validation of the role of HIF-1α in PKM2-mediated macrophage M1 polarization, THP-1 cells were transfected with lentiviral particles containing shPKM2. Next, THP-1 (shPKM2 and shNC) were differentiated into macrophages with 20 ng/mL PMA for 48 h, transfected by siRNA targeting HIF1A, and treated with LPS (1 μg/mL, 24 h) for macrophage M1 polarization. For co-culture experiments between THP-1 and HepaRG cells, THP-1 (shPKM2 and shNC) cells were differentiated and polarized into M1 macrophages, and the supernatant was collected and used to treat HepaRG cells.

### 2.7 Fluorescence activated cell sorting (FACS) analysis

BMDMs from male *PKM2* ^*ΔMAC*^ and *PKM2* ^*FL/FL*^ mice were resuspended and cultured overnight in 6 well plate, and then treated with LPS/IFN-γ for 16 h to induce macrophage M1 polarization. Meanwhile, BMDMs were treated with IL-4 (20 ng/mL, Peprotech, NJ, USA) for 24 h to induce macrophage M2 polarization. Macrophages were collected for flow cytometric analysis using PE anti-mouse F4/80 (565410, BD Biosciences, CA, USA), FITC anti-mouse CD11b (557396, BD Biosciences, CA, USA), BV421 Rat Anti-Mouse CD86 (564198, BD Biosciences, CA, USA), and Alexa Fluor 647 Rat Anti-Mouse CD206 (565250, BD Biosciences, CA, USA) antibodies as previously described (Wang et al., 2022b).

### 2.8 Western blotting

Western blot analysis was performed as previously described (Qu et al., 2024). Briefly, protein of liver tissues and cells were extracted according to a standard protocol, and the protein concentrations were measured respectively. Equal amounts of proteins were loaded onto 10% SDS-PAGE gels, and the gels were transferred to polyvinylidene difluoride (PVDF) membranes (3010040001, Roche). The membranes were blocked with 5% skim milk in TBST and immunoblotted with the indicated primary antibodies at 4°C overnight. The membranes were washed and incubated with a horseradish peroxidase (HRP)-linked secondary antibody for 1 h at room temperature the next day, and the bands were detected using an enhanced chemiluminescence (ECL) kit (170-5061, Bio-Rad, Hercules, CA, USA) and quantified with a ChemiDoc MP Imaging System (Bio-Rad, Hercules, CA, USA). The following primary antibodies were used for Western blotting: p-STAT1 (9167, CST), STAT1 (14994, CST), p-STAT6 (56554, CST), STAT6 (5397, CST), PKM2 (4053, CST), HIF-1α (20960, Proteintech) and β-actin (GB15003, Servicebio).

### 2.9 Real-time quantitative PCR

The qPCR analysis was performed as previously described (Qu et al., 2024). Briefly, the total RNA of livers and cells was isolated by Trizol, and the mRNA expression of targeted gene were normalized to the β-actin mRNA. The following primers were used (5′-3′).

CAG GCG GTG CCT ATG TCT C and CGA TCA CCC CGA AGT TCA GTA G for mouse *Tnfa*; GAA ATG CCA CCT TTT GAC AGT G and TGG ATG CTC TCA TCA GGA CAG for mouse *Il1b*; CTG CAA GAG ACT TCC ATC CAG and AGT GGT ATA GAC AGG TCT GTT GG for mouse *Il6*; TCA ACC GCA ACG AGG AGA AC and CCA CAA ACA GCG ACA CGA CA for mouse *Glut1*; GAG TGG AAT GAA TGT TGC TGG TGT C and CCA GGA TGT GTA GCC TTT GAG TTT G for mouse *Ldha*; GGC TGT ATT CCC CTC CAT CG and CCA GTT GGT AAC AAT GCC ATG T for mouse *Actb*; ACA AGC CTG TAC CCC ATG TT and AAA GTA GAC CTG CCC AGA CT for human *TNFA*; ATG GCA GAA GTA CCT AAG CTC GC and ACA CAA ATT GCA TGG TGA AGT CAG TT for human *IL1B*; ATG AAC TCC TTC TCC ACA AGC GC and GAA GAG CCC TCA GGC TGG ACT G for human *IL6*; CAT CAT GGG CTG GAC ATT GG and CCA CAA AGA TGG TCA CGG TCT G for human *BAX*; GCC CTG TGG ATG ACT GAG TA and ACT TGT GGC TCA GAT AGG CA for human *BCL2*.

### 2.10 Biochemical analyses

ALT activity analysis Kit (BC1555, Solarbio, Beijing, China) was used for detection of serum ALT activity according to the kit instructions.

### 2.11 Enzyme-linked immunosorbent assay (ELISA)

Levels of cytokines including TNF-α (Proteintech, Wuhan, China) and HMGB1 (G-CLONE, Beijing, China) were detected with indicated ELISA kits according to the manufacturer’s instructions. Extracellular levels of glucose and lactate were deter-mined using glucose (Beyotime, Shanghai, China) and lactate assay kit (Beyotime, Shanghai, China) according to the manufacturer’s instructions.

### 2.12 Statistical analyses

Data are expressed as mean ± SD. Statistical analyses were performed using the GraphPad Prism Version 9.0. All data were performed using 2-tailed Student’s t-test or one-way ANOVA. *P* ≤ 0.05 was considered significant difference.

## 3 Results

### 3.1 Macrophage PKM2 was significantly upregulated the in the liver tissues with ALI

**FIGURE 1:**
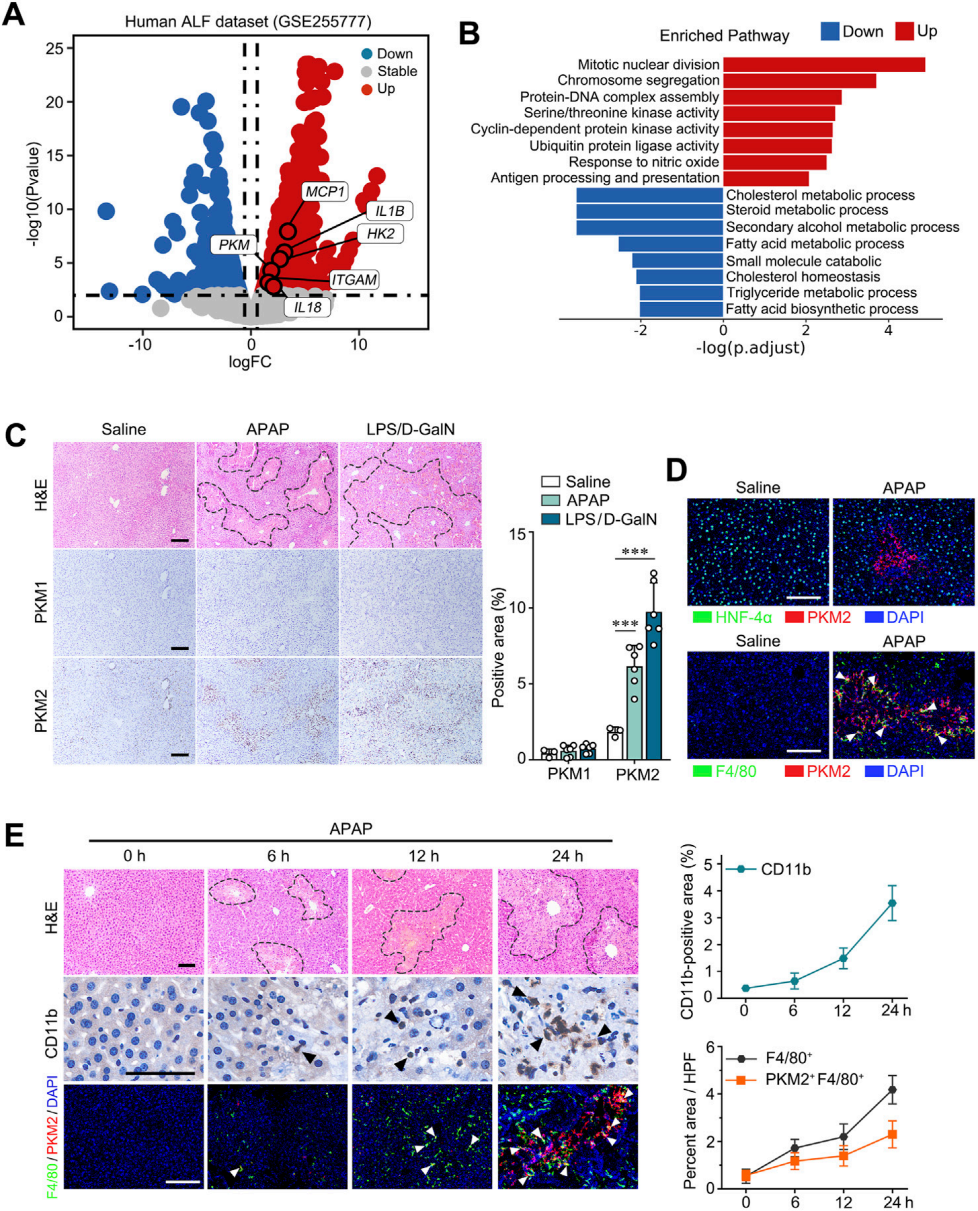
Macrophage PKM2 expression was markedly upregulated in liver tissues with ALI. **(A)** Log ratio–average (M–A) plots showing the changes in gene expression in ALF patients and control individual (GSE255777). **(B)** Gene set enrichment analysis (GSEA) of the DEGs. **(C)** Representative images and quantification of H&E- and immunohistochemistry staining of PKM1 and PKM2 in liver tissues of APAP- or LPS/D-GaIN-induced ALI mice and control mice. **(D)** Dual immunofluorescence staining for PKM2 and HNF-4α **(A)** or F4/80 **(B)** in liver tissues of APAP-induced ALI mice and control mice. **(E)** Representative images of H&E staining, dual immunofluorescence staining for PKM2 and F4/80, and immunohistochemistry staining for CD11b in APAP-induced mouse ALI at the indicated time points with quantification. Scale bar: 100 μm. White arrows indicate PKM2^+^F4/80^+^cells; Necrotic region was enclosed in dotted lines. ***P < 0.001. Error bars depict the standard deviations.

To investigate the role of PKM2 in ALI, we first analyzed human datasets (GSE255777) (Umbaugh et al., 2024) comprising of APAP overdose-induced ALF patient and healthy control. We found that the mRNA level of *PKM* (which encodes PKM1 and PKM2) were significantly enhanced in the liver tissues of ALF patients, accompanied by upregulation of glycolytic genes and pro-inflammatory cytokines (Figure 1A). Meanwhile, we found that pathways involved in the mitotic nuclear division, chromosome segregation and antigen processing and presentation were significantly enriched in ALF liver, possibly attributed to increased hepatocyte regeneration and immune cell activation in response to APAP overdose. (Figure 1B). Immunohistochemistry (IHC) indicated that PKM2, but not PKM1, was significantly upregulated in the liver tissues of murine ALI models induced by acetaminophen (APAP) and lipopolysaccharide/D-galactosamine (LPS/D-GalN), respectively (Figure 1C). From the IHC images, we noticed that PKM2 was particularly upregulated in non-parenchymal cells (NPCs), but not in hepatocytes during ALI progression. Immunofluorescence staining confirmed that PKM2 expression was elevated in NPCs, especially in macrophages within the necrotic region of the ALI livers (Figure 1D). Consistent with what we have observed in NASH livers (Qu et al., 2024), another inflammatory liver condition, we found that most PKM2 was located in the nucleus of F4/80-positive macrophages, suggesting that nuclear translocation of PKM2 is central to hepatic inflammation and the pathogenesis of ALI.

To further analyze the role of macrophage PKM2 in ALI, we assessed the levels of macrophage PKM2 at different time point (0 h, 6 h, 12 h, 24 h) of APAP-induced ALI models (Figure 1E). Interesting, PKM2-positive macrophages were dynamically increased as ALI progressed, accompanied by increased infiltration of monocytes (CD11b). Taken together, these data indicate that macrophage PKM2 may play a key role in regulating hepatic inflammation and ALI pathogenesis.

### 3.2 Macrophage PKM2 knockout alleviated APAP- and LPS/D-GalN-induced ALI

**FIGURE 2:**
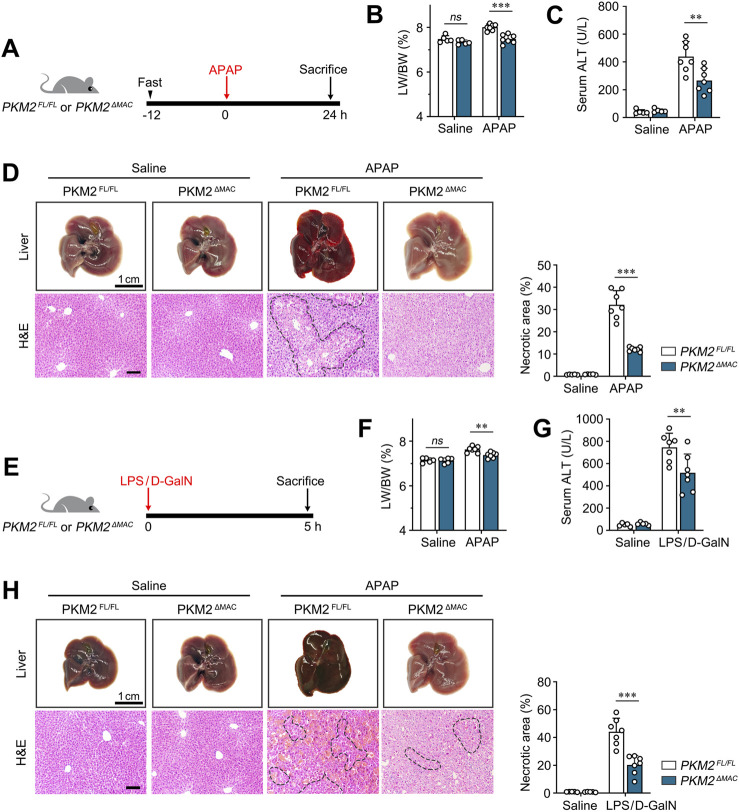
Macrophage PKM2 knockout alleviated APAP- and LPS/D-GalN-induced ALI. **(A)** Scheme diagram depicting *PKM2* ^*ΔMAC*^ and *PKM2* ^*FL/FL*^ mice were challenged with APAP (300 mg/kg) for indicated time. n = 5-7 per group. **(B)** Liver weight to body weight ratio of indicated mice. **(C)** Serum levels of ALT of APAP-induced *PKM2* ^*ΔMAC*^ and *PKM2* ^*FL/FL*^ mice. **(D)** Representative images of livers and H&E staining with quantification of necrosis of *PKM2* ^*ΔMAC*^ and *PKM2* ^*FL/FL*^ mice after APAP induction. **(E)** Scheme diagram depicting *PKM2* ^*ΔMAC*^ and *PKM2* ^*FL/FL*^ mice were challenged with LPS (50 μg/kg) and D-GaIN (700 mg/kg) for indicated time. n = 5-7 per group. **(F)** Liver weight to body weight ratio of indicated mice. **(G)** Serum levels of ALT of LPS/D-GaIN-induced *PKM2* ^*ΔMAC*^ and *PKM2* ^*FL/FL*^ mice. **(H)** Representative images of livers and H&E staining with quantification of necrosis of *PKM2* ^*ΔMAC*^ and *PKM2* ^*FL/FL*^ mice after LPS/D-GaIN induction. Scale bar: 100 μm **P < 0.01; ***P < 0.001. *ns* indicates no significance. Error bars depict the standard deviations. Necrotic region was enclosed in dotted lines.

Next, to determine the function of macrophage PKM2 in ALI, we applied a macrophage PKM2 knockout mice (*PKM2* ^*ΔMAC*^) that was previously generated (Qu et al., 2024). We first examined whether deletion of PKM2 in macrophages protected against APAP-induced ALI. *PKM2* ^*ΔMAC*^ and their control littermates (*PKM2* ^*FL/FL*^) mice were challenged with a sub-lethal dosage of APAP (300 mg/kg) for 24 h (Figure 2A), a model that recapitulates APAP overdose-induced ALI (Kotulkar et al., 2023). As expected, no significant differences were observed in liver weight to body weight ratio (LW/BW) and liver morphology of *PKM2* ^*ΔMAC*^ and *PKM2* ^*FL/FL*^ mice-treated with saline (Figure 2B). After APAP induction, *PKM2* ^*FL/FL*^ mice showed increased serum alanine transaminase (ALT) levels, accompanied by massive myeloid cell infiltration and centrilobular necrosis. Notably, macrophage PKM2 deficiency effectively ameliorated APAP-induced ALI, as demonstrated by improved liver morphology and reduced necrotic area (Figures 2C, D). In addition, the levels of ALT of *PKM2* ^*ΔMAC*^ mice were lower than those of *PKM2* ^*FL/FL*^ mice, implying that macrophage PKM2 ablation reduced hepatic inflammation and hepatocyte injury. Collectively, these results indicate that deletion of PKM2 in macrophage ameliorates APAP-induced ALI.

To further validate the protective effect of macrophage PKM2 knockout on ALI, *PKM2* ^*ΔMAC*^ and *PKM2* ^*FL/FL*^ mice were challenged by LPS/D-GalN (Figure 2E), a more severe ALI model that recapitulate sepsis-induced liver injury (Yang et al., 2023). Consistently, macrophage PKM2 depletion markedly alleviated LPS/D-GalN-induced ALI, as revealed by ameliorated LW/BW, liver morphology, necrotic area and serum ALT (Figures 2F–H).

Taken together, these results indicate that macrophage PKM2 depletion efficiently attenuates ALI in mice.

### 3.3 Macrophage PKM2 deficiency ameliorated hepatic inflammation and hepatocyte apoptosis in ALI mice

**FIGURE 3:**
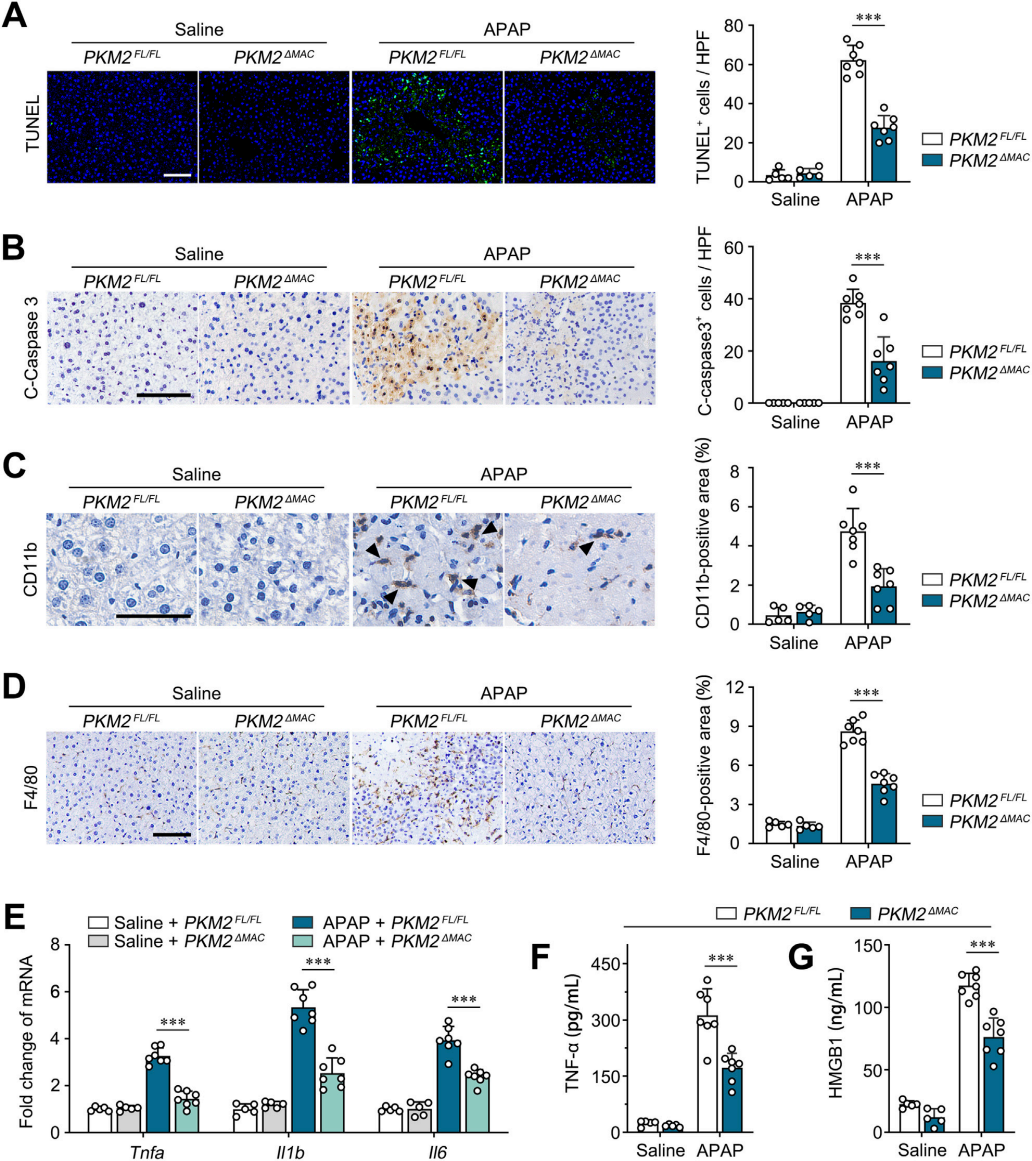
Macrophage PKM2 knockout mice showed ameliorated hepatic inflammation and hepatocyte apoptosis. (**A, B**) Representative images and quantification of TUNEL staining **(A)** and immunohistochemistry staining for Cleaved Caspase 3 **(B)** of *PKM2^ΔMAC^* and *PKM2^FL/FL^* mice after 24 h of APAP induction (300 mg/kg). (**C, D**) Representative images and quantification of immunohistochemistry staining for CD11b **(C)** and F4/80 **(D)** of *PKM2^ΔMAC^* and *PKM2^FL/FL^* mice after 24 h of APAP induction (300 mg/kg). Black arrow indicates CD11b^+^cells. (**E**) The mRNA levels of *Tnfa, Il1b, Il6* in liver tissues of *PKM2^ΔMAC^* and *PKM2^FL/FL^* mice with or without APAP treatment. (**F, G**) The levels of TNF-α **(F)** and HMGB1 **(G)** in serum of *PKM2^ΔMAC^* and *PKM2^FL/FL^* mice with or without APAP treatment. Scale bar: 100 μm. ***P < 0.001. Error bars depict the standard deviations.

The above results demonstrated that macrophage PKM2 knockout resolved APAP- and LPS/GalN-induced liver injury in mice. ALI is characterized by activation of pro-apoptotic pathway and hepatocyte death (Yuan et al., 2023). To validate the restorative effect of macrophage PKM2 knockout on ALI, we assessed the magnitude of hepatocyte apoptosis by TUNEL assay. Here, we found that macrophage PKM2 deficiency significantly attenuated hepatocyte apoptosis, as revealed by reduced TUNEL-positive-cell counts (Figure 3A). Cleaved caspase-3 plays a central role in the execution phase of cell apoptosis, and its expression level was widely applied to evaluate hepatocyte apoptosis (Wu et al., 2014). Consistently, we found that cleaved caspase-3-positive area was markedly diminished in *PKM2* ^*ΔMAC*^ mice, indicating that deletion of PKM2 in macrophages effectively attenuated APAP-induced hepatocyte apoptosis (Figure 3B).

Infiltration of myeloid cell exacerbates hepatic inflammation and hepatocyte apoptosis in ALI. Here, we further analyzed the infiltration of monocytes/macrophages in APAP-induced *PKM2* ^*ΔMAC*^ and *PKM2* ^*FL/FL*^ mice. IHC staining of CD11b and F4/80 showed that compared to control mice, deletion of PKM2 in macrophage significantly inhibited the infiltration of monocytes/macrophages to the injured liver (Figures 3C, D). In addition, we found that the levels of pro-inflammatory genes (*Tnfa*, *Il1b*, *Il6*) and cytokines (TNF-α, HMGB1) were significantly lower in APAP-induced *PKM2* ^*ΔMAC*^ mice compared to *PKM2* ^*FL/FL*^ mice (Figures 3E–G). Collectively, these results demonstrate that deletion of PKM2 in macrophages alleviates ALI by ameliorating hepatic inflammation and hepatocyte apoptosis.

### 3.4 Deletion of PKM2 in macrophages reprogrammed M1 macrophages to M2 macrophages, which thereby attenuated hepatocyte injury

**FIGURE 4:**
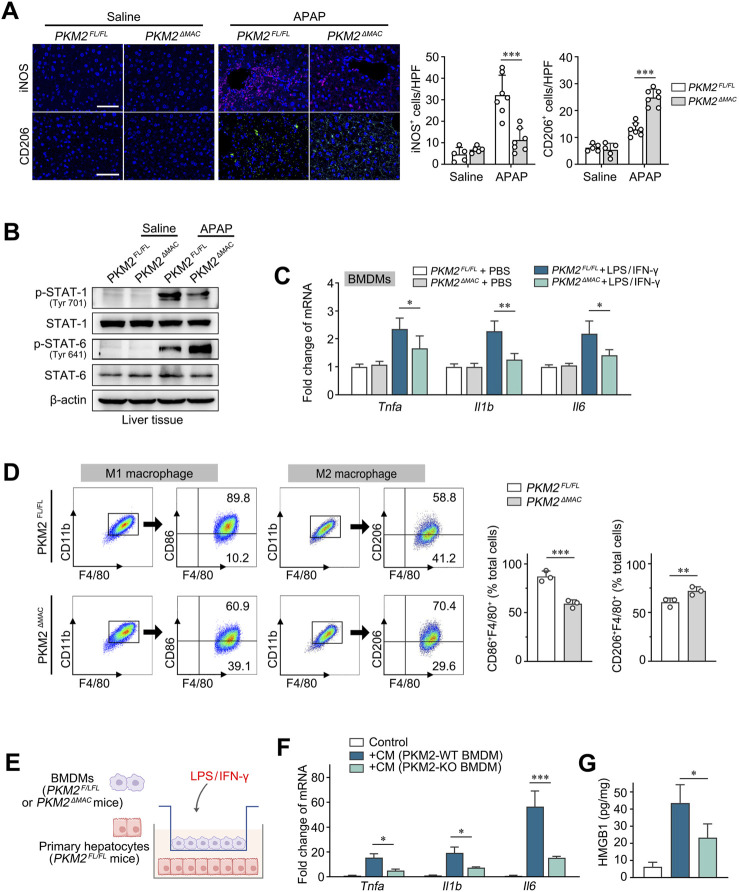
Deletion of PKM2 in macrophages reprogrammed M1 macrophages to M2 macrophages. **(A)** Representative images and quantification of immunofluorescence staining for iNOS and CD206 in the liver tissues of *PKM2* ^*ΔMAC*^ and *PKM2* ^*FL/FL*^ mice after 24 h of APAP induction (300 mg/kg). **(B)** Protein levels of p-STAT1, STAT1, p-STAT6 and STAT6 in liver tissues of *PKM2* ^*ΔMAC*^ and *PKM2* ^*FL/FL*^ mice induced by APAP or saline. **(C)** The mRNA levels of *Tnfa*, *Il1b* and *Il6* in BMDMs of *PKM2* ^*ΔMAC*^ and *PKM2* ^*FL/FL*^ mice treated with or without LPS/IFN-γ. **(D)** Flow cytometry analysis of total macrophages (F4/80^+^CD11b^+^) and M1 (F4/80^+^CD11b^+^CD86^+^) macrophages from BMDMs treated with LPS/IFN-γ, along with analysis of total macrophages (F4/80^+^CD11b^+^) and M2 (F4/80^+^CD11b^+^CD206^+^) macrophages from BMDMs treated with IL-4 *in vitro*. **(E)** Schematic diagram illustrating the co-culture system of BMDMs and primary hepatocytes of indicated mice in the presence of LPS/IFN-γ. **(F)** The mRNA levels of *Tnfa*, *Il1b* and *Il6* of hepatocytes co-cultured with indicated BMDMs. **(G)** The levels of HMGB1 in hepatocytes co-cultured with indicated BMDMs. Scale bar: 100 μm *P < 0.05; **P < 0.01; ***P < 0.001. Error bars depict the standard deviations. BMDMs, bone marrow-derived macrophages.

PKM2 has been recognized as a key regulator of macrophage aerobic glycolysis and pro-inflammatory activation in chronic liver diseases (Qu et al., 2023). In the current study, we found that *PKM2* ^*ΔMAC*^ mice displayed attenuated hepatic inflammation and disease severity in ALI. Herein, we aimed to investigate whether PKM2 worsens ALI by regulating macrophage polarization. Immunofluorescence staining of iNOS (M1 marker) and CD206 (M2 marker) illustrated that the number of M1 macrophages was significantly decreased whereas the number of M2 macrophages was markedly increased in APAP-induced *PKM2* ^*ΔMAC*^ mice compared to *PKM2* ^*FL/FL*^ mice (Figure 4A). Western blot analysis further indicated that phosphorylation of STAT1 (M1 marker) was inhibited while phosphorylation of STAT6 (M2 marker) was enhanced in *PKM2* ^*ΔMAC*^ *mice* compared with control mice (Figure 4B). In addition, in bone marrow-derived macrophages (BMDMs) of *PKM2* ^*ΔMAC*^ and *PKM2* ^*FL/FL*^ mice, we found that the expression of pro-inflammatory genes (*Tnfa*, *Il1b*, *Il6*) was significantly lower in macrophage lacking PKM2 (Figure 4C). Furthermore, BMDMs (F4/80^+^CD11b^+^) from *PKM2* ^*ΔMAC*^ mice treated with LPS/IFN-γ or interleukin-4 (IL-4) showed a decreased ratio of M1 macrophages (CD86^+^) but an increased ratio of M2 macrophages (CD206^+^) (Figure 4D). These results indicate that macrophage PKM2 knockout reprograms macrophages from M1 to M2 phenotype.

To further verify the protective effect of macrophage PKM2 deficiency on hepatocyte apoptosis, we co-cultured BMDMs of *PKM2* ^*ΔMAC*^ and *PKM2* ^*FL/FL*^ mice with primary hepatocytes isolated from *PKM2* ^*FL/FL*^ mice in the presence of LPS/IFN-γ (Figure 4E). Under the stimulation of LPS/IFN-γ, wild-type macrophages significantly induced the levels of pro-inflammatory genes (*Tnfa*, *Il1b*, *Il6*) and cytokines (HMGB1) of hepatocytes, whilst macrophage PKM2 deficiency abolished such differences (Figures 4F, G). In addition, in a co-culture system between human immortalized macrophage (THP-1) and hepatocyte (HepaRG), we found that knockdown of PKM2 in macrophages significantly ameliorated hepatocyte injury and apoptosis, as demonstrated by downregulation of pro-inflammatory genes and pro-apoptotic gene, but increased of anti-apoptotic gene (Supplementary Figure S2).

Collectively, these results indicate that macrophage PKM2 depletion ameliorates hepatocyte injury by reprogramming macrophage polarization.

### 3.5 PKM2 promotes macrophage M1 polarization via HIF-1α signaling

**FIGURE 5:**
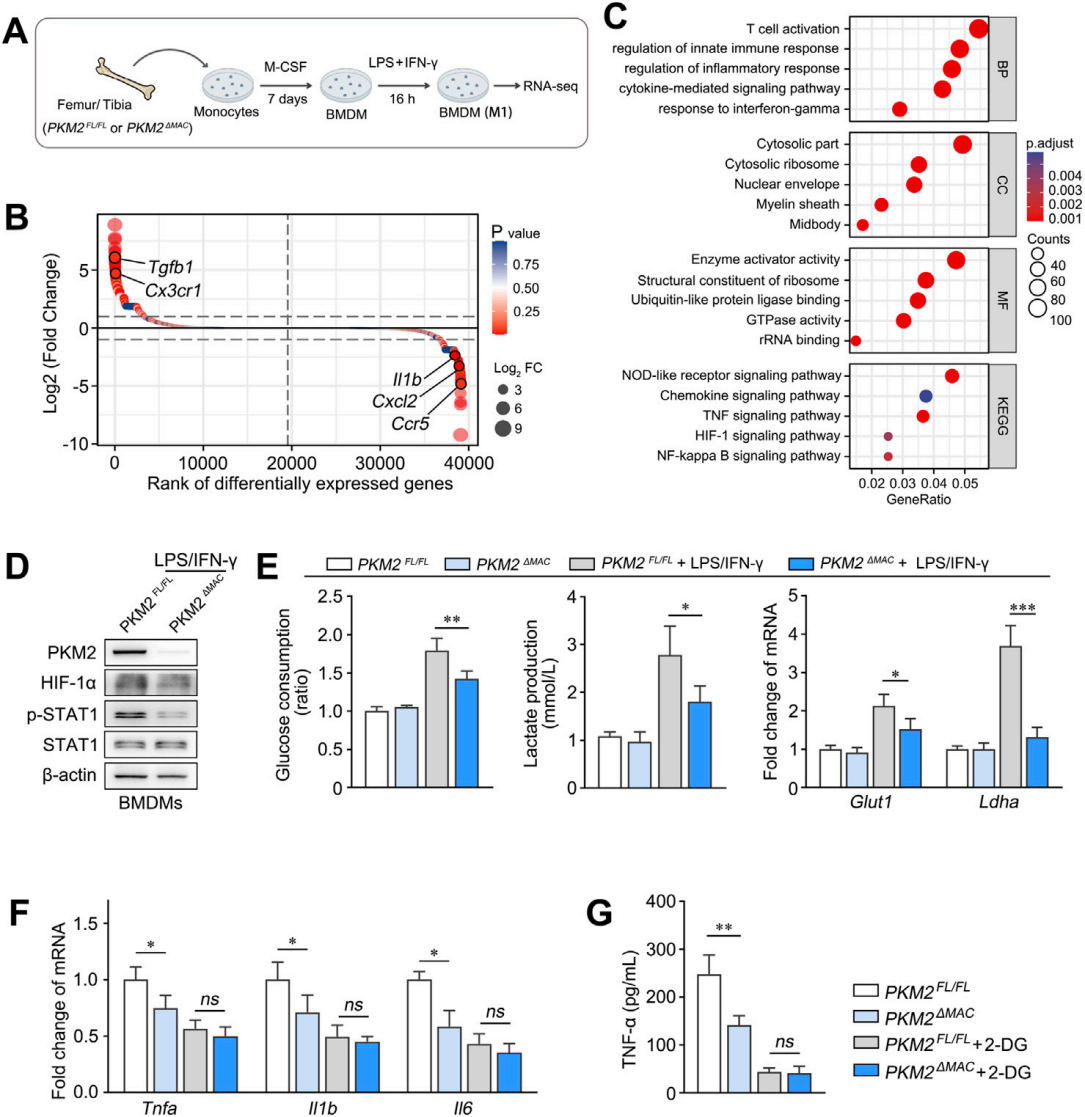
PKM2 promotes macrophage M1 polarization via HIF-1α signaling. (**A**) Schematic diagram illustrating BMDMs of *PKM2^ΔMAC^* and *PKM2^FL/FL^* mice treated with LPS/IFN-γ were subjected to RNA-seq analysis (n = 3 per group). **(B, C)** DEGs **(B)** and GSEA analysis of the DEGs **(C)** between BMDMs of *PKM2^ΔMAC^* and *PKM2^FL/FL^* mice. **(D)** The protein levels of PKM2, HIF-1α, p-STAT1 and STAT1 in BMDMs of *PKM2^ΔMAC^* and *PKM2^FL/FL^* mice treated with LPS/IFN-γ. **(E)** Glucose consumption and lactate production and of BMDMs mRNA levels of Glut1 and Ldha of *PKM2^ΔMAC^* and *PKM2^FL/FL^* mice after LPS/IFN-γ induction. **(F, G)** The mRNA levels of *Tnfa, Il1b* and *Il6* **(F)** and protein levels of TNF-α **(G)** in BMDMs of *PKM2^ΔMAC^* and *PKM2^FL/FL^* mice treated with or without 2-DG (10 mM, 1 h) in the presence of LPS/IFN-γ. *P < 0.05; *P* < 0.01; *P < 0.001; ns indicates no significance. Error bars depict the standard deviations.

To explore the mechanism through which PKM2 regulates macrophage M1 polarization, BMDMs-derived from *PKM2* ^*ΔMAC*^ and *PKM2* ^*FL/FL*^ mice were stimulated by LPS/IFN-γ and then subjected to RNA-seq analysis (Figure 5A). Analysis showed that macrophage PKM2 knockout significantly inhibited pro-inflammatory responses and cytokine production by macrophage (Figures 5B, C). Notably, the expression of IL-1β, a downstream cytokine regulated by HIF-1α signaling, was inhibited attributed to PKM2 deficiency. Studies have reported that PKM2 promotes HIF-1α transactivation, resulting in upregulation of glycolytic genes and macrophage M1 polarization in the context of inflammatory diseases (Palsson-McDermott et al., 2015; Ouyang et al., 2018). Here, we found that HIF-1α activation and STAT1 phosphorylation were significantly inhibited in LPS/IFN-γ-primed BMDMs isolated from *PKM2* ^*ΔMAC*^ mice compared with *PKM2* ^*FL/FL*^ mice (Figure 5D). Glucose consumption, lactate production and the expression of glycolytic genes (*Glut1*, *Ldha*) were also decreased in PKM2-knockouted BMDMs compared with control (Figure 5E). In addition, the expression of pro-inflammatory genes (*Tnfa*, *Il1b*, *Il6*) and cytokines (TNF-α) was lower in macrophage lacking PKM2, whereas the treatment of 2-DG (Inhibitor of glycolysis) effectively abolished such effect (Figures 5F, G).

To further verify the significance of HIF-1α in PKM2-mediated macrophage M1 polarization, we generated THP-1 cells with stable PKM2 knockdown by shRNA, and administered siRNA to silence HIF-1α before LPS treatment. As a result, knockdown of PKM2 significantly downregulated the expression of M1 marker genes (*TNFA*, *IL1B*, *IL6*) in THP-1 cells compared to shNC group, whereas silencing of HIF1A effectively reversed such differences (Supplementary Figure S3).

Collectively, these results demonstrate that PKM2-dependent glycolysis promotes macrophage M1 polarization via HIF-1α signaling.

### 3.6 Therapeutic targeting of PKM2 alleviates ALI progression and prolongs the survival of ALF mice

**FIGURE 6:**
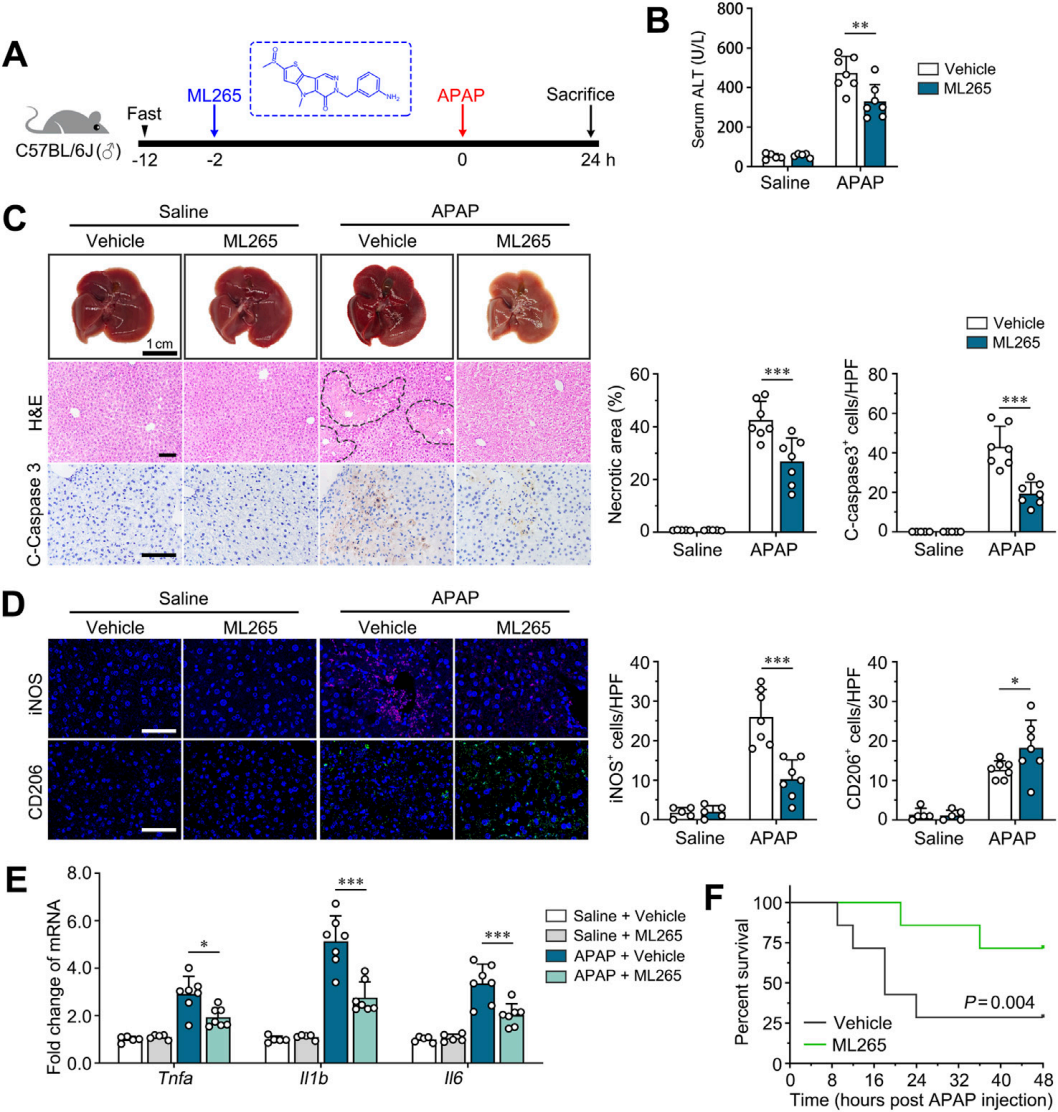
Pharmacological PKM2 antagonist effectively attenuates ALI in mice. **(A)** Scheme diagram depicting C57BL/6J mice pre-treated with ML265 (30 mg/kg), a PKM2 antagonist, were challenged with APAP (300 mg/kg) for indicated time. n = 5-7 per group. **(B)** Serum levels of ALT of APAP-induced mice after ML265 treatment. **(C)** Representative images and quantification of livers, H&E-, IHC of cleaved caspase 3- for ML265- or vehicle-treated mice after 24 h of APAP induction (300 mg/kg). Necrotic region was enclosed in dotted lines. **(D)** Immunofluorescence staining for iNOS and CD206 of ML265- or vehicle-treated mice after 24 hours of APAP induction (300 mg/kg). **(E)** The mRNA levels of *Tnfa*, *Il1b* and *Il6* in ML265- or vehicle-treated mice after 24 h of APAP induction. **(F)** Kaplan-Meier survival curve comparing percent survival of mice pre-treated with ML265 or vehicle after induction of ALF by APAP (750 mg/kg). n = 7 per group. Scale bar: 100 μm. *P < 0.05; **P < 0.01; ***P < 0.001. Error bars depict the standard deviations.

We previously reported that PKM2 antagonist (ML265) effectively ameliorated the progression of many chronic liver diseases, including liver fibrosis and NASH (Qu et al., 2024; Zheng et al., 2020). Here, we aim to evaluate the therapeutic efficacy of ML265 on APAP-induced ALI (Figure 6A).

ML265 treatment did not influence the body weight, liver weight and serum level of ALT of control mice, suggesting that there is no observable hepatotoxicity for ML265 (Figures 6B, C; Supplementary Figure S1). Importantly, ML265 effectively ameliorated APAP-induced ALI compared to control mice, as revealed by improved liver histology, reduced serum ALT, and attenuated hepatocyte apoptosis (Figures 6B, C). Moreover, immunofluorescence staining of iNOS and CD206 demonstrated that ML265 significantly reduced the number of M1 macrophages and increased the number of M2 macrophages, suggesting that the protective effect of ML265 on ALI mice was attributed to repolarization of macrophages (Figure 6D). Consistently, the levels of pro-inflammatory genes (*Tnfa*, *Il1b*, *Il6*) were significantly downregulated in ML265-treated APAP-induced mice (Figure 6E). These results indicate that PKM2 antagonism effectively attenuates ALI progression.

Overdose of APAP is one of the leading causes of acute liver failure (ALF), with a high mortality up to 50% without proper medical manipulation (Yoon et al., 2016). Here, we further examine the therapeutic effect of PKM2 antagonism on ALF mice-induced by lethal dosage of APAP (750 mg/kg) as previous described (Bi et al., 2022). As a result, we found that ML265 significantly prolonged the survival of mice with APAP-induced ALF, accompanied by ameliorated liver histology (Figure 6F; Supplementary Figure S1). These results indicate that PKM2 may serve as a potential therapeutic target for the treatment of ALI and subsequent ALF.

## 4 Discussion

Numerous studies have investigated the role of PKM2 in the progression of chronic liver diseases, including liver fibrosis (Rao et al., 2022), fatty liver diseases (Dong et al., 2024) and even liver cancers (Hou et al., 2020). However, whether PKM2 contributes to ALI pathogenesis remains largely unexplored. In the present study, we analyzed the expression pattern, function and regulatory mechanism of PKM2 in the progression of ALI, and found that PKM2 exacerbated ALI by inducing macrophage M1 polarization. Furthermore, we demonstrated that PKM2 may serve as a therapeutic target for the treatment of ALI.

PKM2 is primarily upregulated in non-hepatocyte cells, especially in macrophages, in the context of ALI. This finding aligns with our previous report that PKM2 is mainly expressed by non-parenchymal cells (NPCs) in abnormal liver condition (Qu et al., 2023). Interestingly, another study reported that PKM2-dependent glycolysis is required for the activation of invariant natural killer T cells in response to ALI (Aguiar et al., 2023). Indeed, PKM2 is involved in the activation of many NPCs, including macrophages (Xie et al., 2016), neutrophils (Dhanesha et al., 2022), T cells (Moreno-Fernandez et al., 2021), and stellate cells (Zheng et al., 2020). The potential role of PKM2 in other NPCs in ALI requires further investigation.

We have previously reported that deletion of PKM2 in macrophages attenuates hepatic inflammation and NASH fibrosis progression, accompanied by reduced pro-inflammatory Ly6C^high^ macrophages infiltration (Qu et al., 2024). In accordance with our previous study, we found that macrophage PKM2 deficient mice are less susceptible to hepatic inflammation and ALI, with reduced iNOS-positive macrophages and increased CD206-positive macrophages. Although the effect of PKM2 knockout on macrophage polarization is comparable between NASH and ALI, the infiltration of macrophages is more robust and rapid in ALI. Although we found that the expression of *Cxcl2* and *Ccr5* were lower in PKM2-KO macrophages (Figure 6B), whether macrophage PKM2 participate in regulation of chemokines release and recruitment of myeloid cells during ALI progression remains to be explored.

PKM2 has been recognized as a key player in metabolic reprogramming and activation of macrophages. On one hand, enzymatic hyperactive PKM2 tetramer speed up glycolytic flux to fuel aerobic glycolysis, lactate production and lactylation (Pan et al., 2022). On the other hand, nuclear translocation of PKM2 dimer binds with transcription factor, including STAT3 and HIF-1α, to induce the transcription of downstream glycolytic genes (Ouyang et al., 2018; Hou et al., 2020). In the current study, we found that PKM2 is mainly expressed by macrophages in ALI livers. Interestingly, we observed that most PKM2 is localized in the nucleus of macrophages, suggesting that PKM2 dimer, but not tetramer, may play a more important role in ALI progression. Consistently, we found that HIF-1α signaling and glycolysis were markedly inhibited in PKM2-deficient macrophages in APAP-induced ALI and LPS/IFN-γ-induced BMDMs. These results illustrate that PKM2 induces macrophage glycolysis and M1 polarization via HIF-1α signaling.

PKM2 targeting compounds are mainly characterized into PKM2 enzymatic inhibitor (limit PKM2 tetramer formation) and PKM2 antagonist (limit PKM2 nuclear translocation). Given that nuclear translocation of PKM2 is central to macrophage polarization and ALI progression, we choose PKM2 antagonist as therapeutic intervention and found that PKM2 antagonism efficiently ameliorates APAP-induced ALI by reprogramming macrophage polarization. Recently, therapeutic modulation of macrophage polarization in the treatment of inflammatory diseases, including ALF (Xie and Ouyang, 2023), liver fibrosis (Tacke, 2017), osteoarthritis (Wang et al., 2023) and even cancer (Chen et al., 2023), have caught increasing attention. The current study highlights the translational potential of PKM2 as a druggable target for macrophage repolarization and ALI, adding value to the preclinical evidence of PKM2 antagonism on combating inflammatory liver diseases.

## Data Availability

The original contributions presented in the study are publicly available. This data can be found at the NCBI (GEO) repository, accession number GSE294508.
