# Supplementary material for: Macrophage PKM2 depletion ameliorates hepatic inflammation and acute liver injury in mice
Source: Front Pharmacol. 2025 Apr 25;16:1546045. doi: 10.3389/fphar.2025.1546045 (PMC12062095; doi:10.3389/fphar.2025.1546045)
Supplement: Supplementary file 1 [file DataSheet1.docx]

**Supporting Information**

**Macrophage PKM2 depletion ameliorates hepatic inflammation and acute liver injury in mice**

Ziwei Kang^1,2^, Ruoyan Xie^2^, Yiming Cui^2^, Zhiwei Chen^3^, Jincheng Li^4^, Jinyu Lv^2^, Weijia Ye^4^, Peixin Zhao^2^, Keke Zhang^1,2*^, Jian Hong^1,2*^, Hengdong Qu^1,2*^

^1^*State Key Laboratory of Bioactive Molecules and Druggability Assessment, Jinan University, Guangzhou, China.*

^2^*Department of Pathophysiology, School of Medicine, Jinan University, Guangzhou, China.*

^3^*Department of Hepatobiliary Surgery, The First Affiliated Hospital, Jinan University, Guangzhou, China.*

^4^*Center of Hepato-Pancreato-Biliary Surgery, the First Affiliated Hospital, Sun Yat-sen University, Guangzhou, China.*

*Corresponding authors.

**Contact Information:**

Dr. **Hengdong Qu**, State Key Laboratory of Bioactive Molecules and Druggability Assessment, Jinan University; E-mail: quhengdong@gmail.com.

Dr. **Jian Hong**, State Key Laboratory of Bioactive Molecules and Druggability Assessment, Jinan University; E-mail: [hongjian7@hotmail.com](mailto:hongjian7@hotmail.com).

Dr. **Keke Zhang**, Department of Pathophysiology, School of Medicine, Jinan University; E-mail: kkzhang@jnu.edu.cn.

**Supplementary Figure. S1**


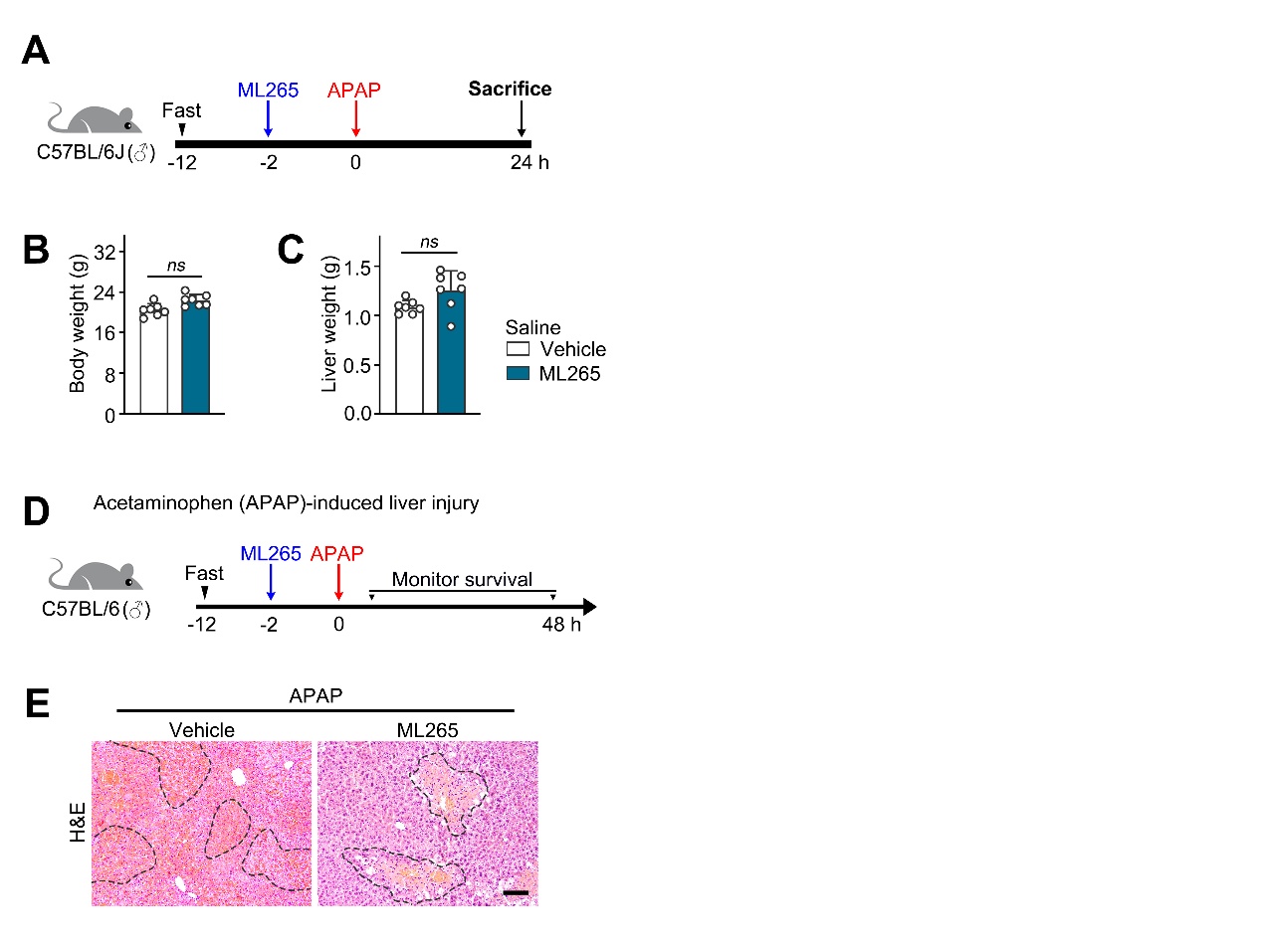


**Figure S1. ML265 treatment efficiently ameliorated APAP-induced ALI without causing significant hepatocoxicity.** (**A**) Scheme diagram depicting C57BL/6J mice pre-treated with ML265 (30 mg/kg), a PKM2 antagonist, were challenged with APAP (300 mg/kg) for indicated time. (**B-C**) Body weight (B) and liver weight (C) of indicated mice. (**D**) Scheme diagram depicting C57BL/6J mice pre-treated with ML265 or vehicle were challenged by APAP (750 mg/kg). (**E**) Representative images of H&E staining of liver tissues of indicated mice after 48 h of APAP induction. Scale bar: 100 μm. ns indicates no significance. Error bars depict the standard deviations.


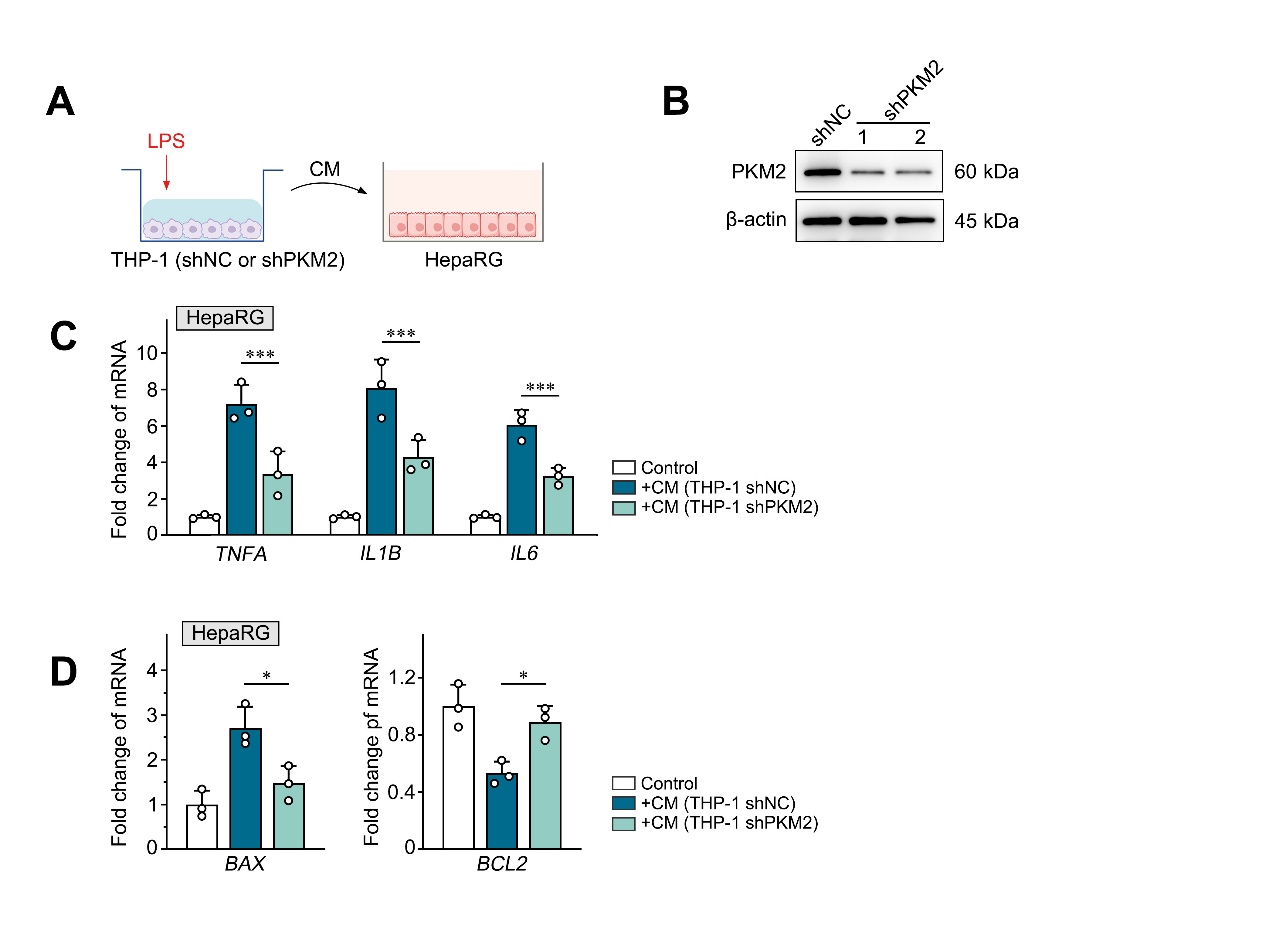


**Figure S2. Knockdown of PKM2 in macrophages ameliorated hepatocyte inflammatory response and apoptosis.** (**A**) Scheme diagram depicting THP-1 (shNC or shPKM2) was differentiated by PMA (20 ng/mL, 48 h) and treated with LPS (1 μg/kg, 24 h), then the conditional medium (CM) were collected and co-cultured with HepaRG cells. (**B**) Validation of knockdown efficacy of PKM2 in shNC- and shPKM2-transfected THP-1 cells. (**C**) mRNA levels of pro-inflammatory genes in CM-treated HepaRG cells. of indicated mice. (**D**) mRNA levels of pro-apoptotic and anti-apoptotic genes in CM-treated HepaRG cells. *P < 0.05; ***P < 0.001. Error bars depict the standard deviations.


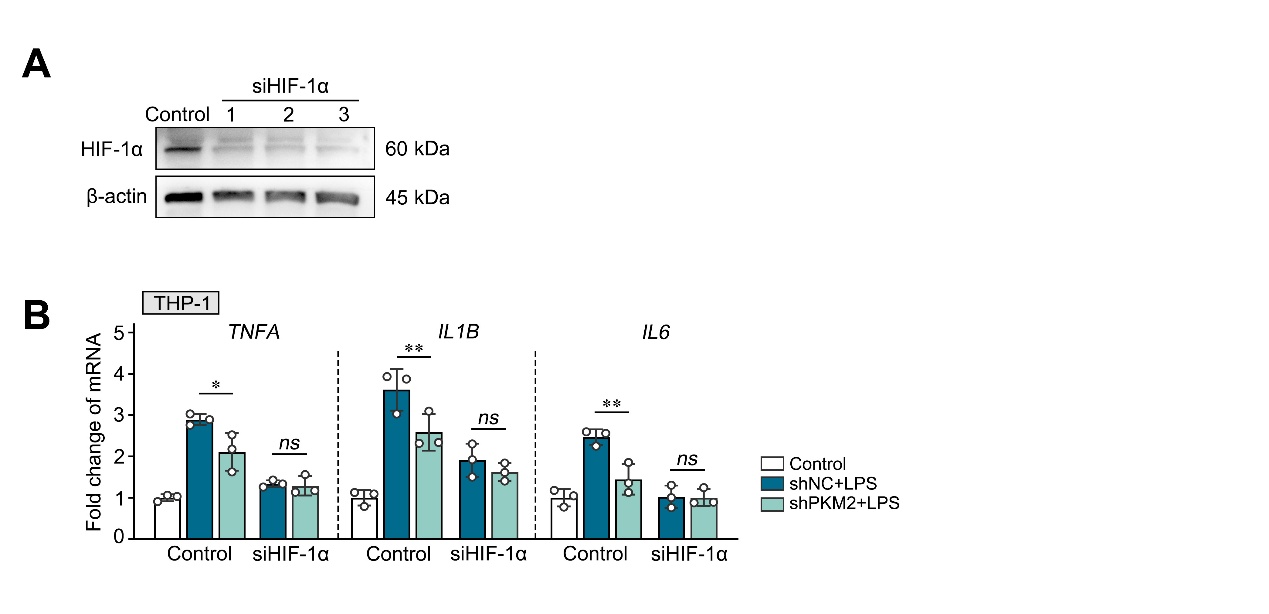


**Figure S3. PKM2-mediated macrophage M1 polarization is HIF-1α dependent.** (**A**) Knockdown efficacy of siRNA-targeting HIF1A in THP-1 cells. (**B**) mRNA levels of pro-inflammatory genes in THP-1 cells (shNC or shPKM2) with siHIF1A transfection. *P < 0.05; **P < 0.01. ns indicates no significance. Error bars depict the standard deviations.
